# The Potential Role of Super Spread Events in SARS-COV-2 Pandemic; a Narrative Review

**Published:** 2020-09-21

**Authors:** Anthony M. Kyriakopoulos, Apostolis Papaefthymiou, Nikolaos Georgilas, Michael Doulberis, Jannis Kountouras

**Affiliations:** 1 Department of Research and Development, Nasco AD Biotechnology Laboratory, Piraeus 18536, Greece.; 2 Department of Gastroenterology, University Hospital of Larisa, Larisa 41110, Greece.; 3 Department of Internal Medicine, Second Medical Clinic, Ippokration Hospital, Aristotle University of Thessaloniki, Thessaloniki, 54642 Macedonia, Greece.; 4 Department of Nephrology, Agios Pavlos Hospital of Thessaloniki, Thessaloniki 55134, Macedonia, Greece.; 5 Division of Gastroenterology and Hepatology, University Medical Department Kantonsspital Aarau, Aarau 5001, Switzerland.

**Keywords:** Pandemics, epidemics, coronavirus, severe acute respiratory syndrome coronavirus 2, disease outbreaks, cost of illness, mass vaccination

## Abstract

Coronaviruses, members of *Coronaviridae* family, cause extensive epidemics of vast diseases like severe acute respiratory syndrome (SARS) and Coronavirus Disease-19 (COVID-19) in animals and humans. Super spread events (SSEs) potentiate early outbreak of the disease and its constant spread in later stages. Viral recombination events within species and across hosts lead to natural selection based on advanced infectivity and resistance. In this review, the importance of containment of SSEs was investigated with emphasis on stopping COVID-19 spread and its socio-economic consequences. A comprehensive search was conducted among literature available in multiple electronic sources to find articles that addressed the “potential role of SSEs on severe acute respiratory syndrome coronavirus 2 (SARS-COV-2) pandemic” and were published before 20^th^ of August 2020. Overall, ninety-eight articles were found eligible and reviewed. Specific screening strategies within potential super spreading host groups can also help to efficiently manage severe acute respiratory syndrome coronavirus 2 (SARS-COV-2) epidemics, in contrast to the partially effective general restriction measures. The effect of SSEs on previous SARS epidemics has been documented in detail. However, the respective potential impact of SSEs on SARS-COV-2 outbreak is composed and presented in the current review, thereby implying the warranted effort required for effective SSE preventive strategies, which may lead to overt global community health benefits. This is crucial for SARS-COV-2 pandemic containment as the vaccine(s) development process will take considerable time to safely establish its potential usefulness for future clinical usage.

## Introduction

Severe acute respiratory syndrome (SARS) has periodically emerged as epidemics and its natural history could be utilized as a “compass” to comprehend and manage the current pandemic of SARS-COV-2. SARS-COV-2 the etiologic agent of the novel coronavirus disease 2019 (COVID-19), belongs to RNA coronavirus family (*Coronaviridae*) and is a zoonotic coronavirus that has crossed species barriers to infect human (1-3). The initially investigated strains of COVID-19 exhibited low potential for transmissibility and infectivity, similar to SARS coronavirus (SARS-COV) (1-4). Moreover, SARS epidemic was potentiated due to super spread events (SSEs), which led to unexpected elevation of the basic reproduction numbers as calculated via associated epidemiology equations (5). Specifically, SSEs resulted from secondary contacts of carriers (6, 7). Infected individuals, as mediators of SSEs, represent the initial cluster of viral transmission (8); thus, inducing an exponential secondary contamination (4). Although the prediction and subsequently the prevention of SSEs seems to be complicated, the virus, host, environmental, and mass behaviors determine relative approaches to prevent and control SSEs; core community health programs can inhibit and decrease the incidence and the effect of SSEs (9). Nevertheless, horizontal austerity measures, such as recommending or compelling individuals to self-isolate at home, which might cause serious social and psychological burden, and quarantine, also leading to loss of income due to social distancing, are associated with negative psychological and religious effects, which can be long lasting (10), thereby leading to serious instability of the global society. Prolonged social isolation and loneliness are associated with increased mortality (11). 

Currently, limited piece of information exists regarding the effect of SSEs on coronavirus epidemics. The aim of this narrative review is to mainly focus on the potential impact of SSEs on large outbreaks of coronavirus. The development of an emergency SARS-COV-2 vaccine has its potential usefulness and/or limitations and may result in severe health outcomes, which prompts better screening for SSEs in order to control coronavirus pandemics. 

## Methods

**2.1 Methodological approach**

To avoid, in most respects, literature selection bias (12), multiple electronic sources: Medline/PubMed, SciFinder, Science Direct and Goggle Scholar as well as ResearchGate and General (Google) were investigated via queries with a non-restricted time frame reaching the 20^th^ of August 2020. Initial investigation of SSEs and SARS, SSEs and MERS, and SSEs and COVID-19, gave narrative results from PubMed. The selected literature, which is included in the study, is presented in table 1. Same items were also searched in all other mentioned sources. The scope of the study was not only to investigate the transmission of SARS-COV-2 due to SSEs, its comparison with SARS-COV-1 and MERS-COV, but also to assess the general global impact due to SSEs by COVID-19. Therefore, further literature investigation was performed using the same electronic sources. Further investigation was made on: a) the prevention of SSEs by coronaviruses causing SAR-1, MERS and COVID-19, b) the socio-economic relation of SARS-COV-1, MERS and COVID-19 due to SSEs, c) the austerity caused by SSEs of COVID-19, and d) the relation of SSEs containment to future vaccination programs. For further investigation, the following items were searched: “SARS, MERS and COVID-19 Epidemic Prevention”, “SARS MERS and COVID-19 Infectivity and Pathogenicity”, “Coronavirus SSE Prevention”, SSE Coronavirus Crisis and Socio-economics”, “Holy Cup Religion and Transmission of Pathogens and SSEs”, and “Coronavirus Immunity and Vaccination”. 

**2.2 Selection process**

**2.2.1 Screening Process and Eligibility Criteria**

Studies providing an adequate determination of an SSE related to SARS, MERS and COVID-19 were primarily screened and selected by two reviewers (authors) blinded to one another. The results were thereafter cross-matched and duplicates were removed. Based on this primary search, the socio-economic impact of coronavirus, produced by SSEs, was extrapolated by two other reviewers (authors). Following this initial selection stage, further screening was performed by all reviewers, using the previously described search items to identify parameters determining the global impact of COVID-19 due to SSEs. Identified parameters included the global impact of immunity and vaccination, the holy cup and religion transmission, and the austerity caused by COVID-19 and other coronavirus epidemics due to restrictions applied. All search results were cross-matched to remove duplicates and thereafter, exclusion and inclusion criteria were applied. 

**2.2.2 Exclusion and Inclusion Criteria**

After removing the duplicates, review was conducted on titles and abstracts. Also, a decision was made to remove “news press opinions”. Computational model methodologies producing contradictory results, studies with wrong interpretation of SSEs, and studies with non-clear-cut results were also removed. Studies using the interpretation “a super spreading individual, known as the index case, produces a cluster of SARS, MERS, and COVID-19 secondary infections” were included. A second exclusion criterion was applied. In this stage, peer reviewed literature of recent dates, studies assessing SARS, MERS, and COVID-19 epidemiology measures, studies on COVID-19 restriction measures producing social and economic austerity, articles discussing the perspective for future vaccination and population immunity, and finally genetic studies on coronaviruses causing SARS, MERS, and COVID-19. 

## Results

**Figure 1:**
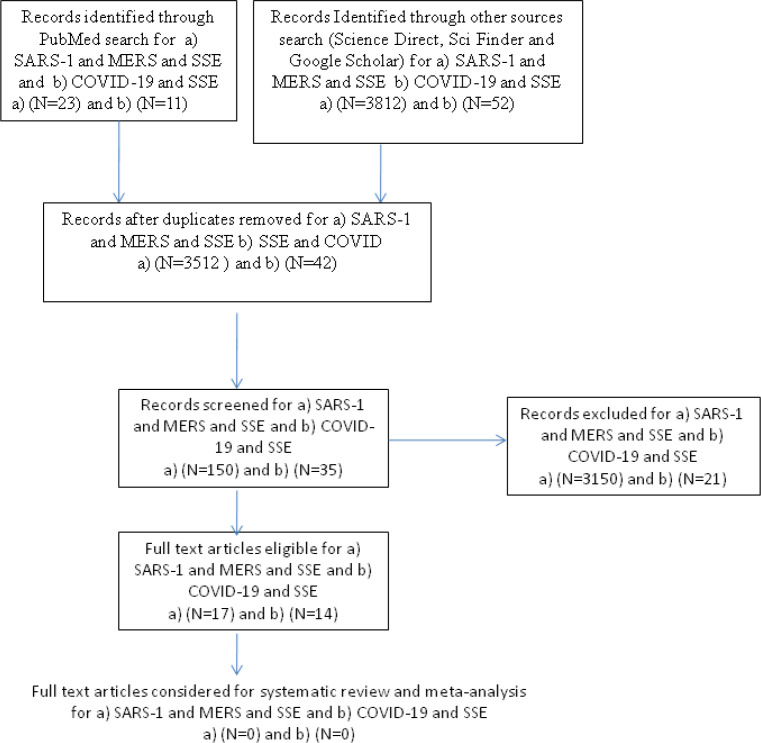
Method followed for PubMed search and literature selection regarding SSE in relation to a) SARS-1, MERS and b) COVID-19 outbreaks

By following the described methodology, on Medline/PubMed: a) 23 articles were found on SARS and MERS and SSE, and b) 11 articles were found on COVID-19 and SSEs. Out of: a) 13 of the 23 articles on SARS and MERS and SSE, and b) 7 out of the 11 articles on COVID-19 and SSE were deemed relevant hits. After applying the exclusion criteria, 12 articles from the first category, and 4 from the second category were included in the study. Suitable articles found by searching, which were selected and reviewed for each part, are illustrated in figure 1. Further investigation in all other electronic sources described, using the same methodology, increased the number of the included literature to a) 17 and b) 14, for their respective categories of search. Studies included from PubMed in these categories of searches are briefly described and listed in table 1. Further, assessing the general global impact of SSEs related to COVID-19, using all the mentioned sources, via the same methodology, led to the inclusion of a) 10 articles related to genetic analysis of SARS-COV-1 and MERS-COV and SARS-COV-2, b) 5 articles related to super spread events, c) 2 articles related to austerity, d) 18 articles related to infectivity and pathogenicity of SARS, MERS and COVID-19, e) 17 articles related to prevention of SSEs concerning human coronaviruses, f) 9 articles related to socio-economic impact, and g) 9 articles related to immunity and future vaccination. Table 2 illustrates the initial numbers of hits using all search items in all sources, and the final number of articles reviewed in each category. 

## Discussion

**4.1 Insights to SSEs**

The involvement of SSEs in SARS extensive outbreaks (1, 4, 5, 13-17), necessitates urgent elucidation as global tranquility is disturbed by COVID-19 pandemic. Epidemiological research has proposed that the outbreak was related to a seafood market in Wuhan (Hubei, China), underlining the ongoing risk of viral transmission from animals to induce severe diseases in humans. Metagenomic RNA sequencing of bronchoalveolar lavage fluid from a patient with pneumonia identified a novel RNA virus strain from the Coronaviridae family  (called SARS-COV-2); and phylogenetic analysis (by introducing the widely used *in silico* protein screening) (18-21) of the complete viral genome (29,903 nucleotides) disclosed that the virus was most closely connected (89.1% nucleotide similarity) with a group of SARS-like coronaviruses (genus *Betacoronavirus*, subgenus *Sarbecovirus*) formerly isolated from bats in China (18-22).

Insights from previous reports by Menachery et al. (23) (Menachery *et al.*, 2015), pointed out that the 2002-2003 emergence of SARS-CoV introduced the possibility of viruses of animal origin causing epidemics in human populations. Conclusions from their study revealed, as previous studies had demonstrated (1, 5, 13, 15), that closely related SARS-like viral genes were traceable in Chinese bat populations. Authors claimed that these viruses were capable of infecting humans, by selective adaptations or adjustments, and thereby, causing a new epidemic (23). Enhancement of virulence is also attributed to these adaptations due to acquisition of spike protein via adaptive mutations (24). 

**Figure 2:**
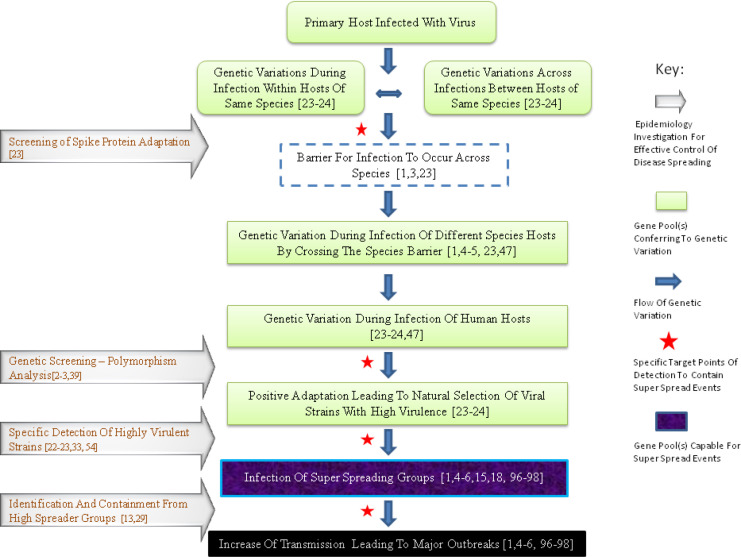
Flow of genetic variation of coronaviruses leading to increase of virulence and pathogenicity; epidemiologic steps for specific and targeted diagnosis to prevent super spread events. Blue arrows point to the flow of genetic variation across gene pools where genetic variation occurs, i.e. a) between hosts of the same species, b) between hosts of different species by crossing the species barrier, spreading to c) humans and subsequently to super spreader individuals, where disease transmission is potentiated. Grey arrows point to specific identifications that can lead to effective interventions with the potential to control the disease spread

Continuous viral random mutations are possible through intermediate host transmission, until a deadly virus develops, as illustrated in Figure 2. Recent evidence revealed that recombination within intermediate hosts has contributed to development of SARS-COV-2 (1, 24). Asian outdoor markets could constitute the ideal places for continuous viral mutation exchanges (25). As presented in Table 3, the best way to circumvent continuous virus production is targeted surveillance; to at least stop the overspreading by SSEs (2, 3, 22, 26). This has also been proposed by Menachery et al. (23).

**4.2 SARS epidemics and SARS-COV-2 pandemic**

SARS-COV-2 is accountable for the unprecedented COVID-19 pandemic (27), and the interplaying mechanisms involved in the pathophysiology of COVID-19 include SARS-COV-2 virulence, host immune response, and complex inflammatory reactions (28). Emerging data, also, imply that the reservoirs of SARS-COV-1 infection may be similar to COVID-19 (1, 4, 5, 13, 29), as remarkable similarities exist between SARS and swine acute diarrhea syndrome (SADS) in topographical, temporal, environmental and etiological backgrounds. However, the increasing coronavirus variety and spread in bats were recognized as a potential target to diminish future epidemics that might impend livestock, community health, and financial progress (30). Probably, identification of animal and insect vectors that transmit the disease, identification and control of alternative routes of transmission like fecal-oral route, and identification of super spreader patient groups could help minimize the epidemiological extent compared to the one observed for SARS-COV-2 infection worldwide. Lessons from SARS epidemic taught us that the key to control is minimizing the time from the diagnosis of infection to prompt hospital isolation and diminishing the probability of another SSE (5).

**4.3 The 20/80 rule as applied to SARS**

The typically recognized 20–80 rule or the so-called “Pareto rule”, states that 20% of efforts lead to 80% of results (31). More specifically, this comprises a principally convenient state when tackling infectious diseases and is applied to investigate infection transmission, and initially among cattle farms. In this regard, Woolhouse et al. (17) reported that targeted actions concerning disease control and prevention in 20% of the farms that mainly supplied the basic reproduction number (R_o_) decreased spread by 80% (32). Focusing on the COVID-19 virus, R_o_ is a sign of virus transmissibility, denoting the average figure of novel infections caused by an infectious individual in a totally naïve population. For *R*_0 _> 1, the number of infected people tends to increase, whereas for *R*_0 _< 1, transmission is likely to stop; R_o_ represents a chief model in the epidemics, signifying the risk of an infectious mediator with regard to epidemic spread (33). Recent data indicate that the estimated mean R_o_ for COVID-19 is almost 3.28, with a median of 2.79 and the interquartile range (IQR) of 1.16, which is substantially higher than WHO’s estimation of 1.95. However, due to biased methodology, R_o_ for COVID-19 is expected to be about 2–3, which is approximately consistent with the WHO estimate (33). SSEs appear to be a main limitation of the R_o_ concept. R_o_, when calculated as a mean or median value, does not include the heterogeneity of transmission between infected individuals (4); two infective agents with equal R_0_ estimates might have noticeably diverse patterns of transmission. Moreover, the goal of a Health Care System is to achieve R_o_ <1, which is probably only phenomenally feasible in certain conditions without scheduled prevention, recognition, and response to SSEs (9). Naturally, epidemics follow the aforementioned 20 / 80 rule (17). Specifically, in human population, due to heterogeneous exposure to infectious agent, the 20% core population may transmit the disease, widely. For SARS, the rate might have been even lower than 20% (4). The increased infectious potential of a small population subgroup seems to be related to immunodeficiency, such as in hemodialysis, cancer, immunosuppressive therapies (4, 5, 15, 34). Additionally, facilitation of disease spread and transmission due to vector exposure has been investigated in relation to cockroaches (35). Possible mechanical transportation by rats and cat (13, 36) and air transmission (37) in SARS-COV-1 have also been studied. Other animals capable of being SARS-COV-2 carriers (excluding mice and rats), like pigs, ferrets, cats, and non-human primates have recently been introduced (3), and contamination of sewage with SARS-COV-2, has probably preceded COVID-19 outbreak in France (29). All these agents may contribute to a minimum of 80% of the total transmission potential (17), maybe even more (4, 5). Table 4 displays possible super spreader groups; thus, indicating screening targets to prevent SSEs. SARS epidemic taught us that control programs were inefficient in controlling the epidemic within a population, and failed to identify and provide a targeted infection diagnosis in groups causing potential SSEs (5, 17). On the other hand, SARS-COV-2 having the ability to cause a pandemic rather than an epidemic, resulted in an increased number of cases and deaths; albeit having a lower mortality rate than SARS coronavirus (2). SSEs during COVID-19 may involve not only one city, but also a whole country or many countries, requiring investigation of their effects on a national or international level (2, 38, 39). 

**4.4 Prevention of SSEs**

Preventing and decreasing COVID-19-related SSEs necessitates the decryption of the mechanism through which SARS-COV-2 spreads through super spreader individuals, for example within healthcare facilities (7, 9). Healthcare facilities are essential for prevention and control of SSEs (9). SSE prevention may enable us to even overcome initial low COVID-19 virus infectiveness. The capability of the virus to produce SSEs troubles the epidemiological attempts to restrict viral spread only by isolating individuals at high risk and performing obsolete isolation at home for the general population as carried out in countries such as Greece (5). During the SARS epidemic in China (Beijing) and Singapore, the vast majority of infected individuals were barely infective and only 6% of the population was highly infectious, in contrast to many published SARS models (4, 5). Other ways of potential coronavirus transmission between hosts may provide explanations for enormous outbreaks (16). It should not be disregarded that coronaviruses cause both respiratory and intestinal infections and share common evolutionary roots with hepatitis viruses (40, 41). Passing the cross-species barrier and genetic adaptation within hosts may promote virulence of coronaviruses in humans (14). This, prompts to specifically identify potential super spreader groups within populations through targeted diagnosis. Some of these groups are listed in Table 2. For this purpose, a usual infection must be distinguished from a super spread infection (4, 5). During SARS epidemic, the coronavirus infectiousness mostly occurred in the late stages of infection (5, 17), whereas in COVID-19, viruses are transmitted even in pre-symptomatic stages (42). As with Influenza A virus subtype H1N1 transmission (43), accurate diagnosis of COVID-19 in potentially asymptomatic super spreaders may help contain the magnitude of large outbreaks (44). 

In the case of Diamond Princess Cruise ship, an early-assessed R_0_ of 14.8 (≈4 times higher than the R_0_ in the epicenter of the outbreak in Wuhan, China) was decreased to an assessed effective R_o_ of 1.78 following on-board isolation and quarantine processes (45). Similarly, in China (Wuhan) the application of non-pharmaceutical interventions in the society, including a cordon sanitaire of the town; interruption of community transport, school, and most employment; and termination of all community events decreased the R_o_ from 3.86 to 0.32 over a 5-week period (46). Nevertheless, these strategies could not be maintained.

Emerging research evidence (29) regarding sewage contamination that preceded Paris COVID-19 epidemic is pointing to the reports of 2003 from the health department of Hong Kong (35, 36), the noble work by Ng (13), and urge for extensive environmental monitoring (29, 37) to prevent future COVID-19 relapses. However, the flow of genetic variation may be even more complex as illustrated in Figure 2. Therefore, advanced clinical and laboratory monitoring is required to prevent SSEs and thereafter, new coronavirus epidemics. Assembly of key functions and screening techniques of reference centers is presented in Table 3. Newer therapeutic agents and protocol applications are promising (47), although probably carrying the possibility of resistance state (48). First, these also require specific diagnostic and surveillance strategies to overcome any unknown adverse epidemiology consequences (48). Inhibiting wild meat markets and related consumption of wild meat by creating vivid campaigns could be a critical for interrupting the introduction of coronaviruses crossing from animals to the human population, as was the case for SARS (1, 4, 5) and Middle East respiratory syndrome (MERS) (49) epidemics, and probably now for COVID-19 pandemic (1-3). Furthermore, the food production process requires radical reconsideration, concerning the industrial environment of current food production and serious violations of natural ecosystems (50). Current industrial procedures for preparing food increasingly favor conditions where viral evolution produces new mutations and increased rates of mutations (25), thus raising the probability of new and more infectious viral strains. In SARS and MERS epidemics, the role of SSEs in vigorously distributing the epidemics has been substantially proven (51-55). The new COVID-19 epidemiology evidence also adequately highlights the important role of SSEs in homeland of China (21, 56, 57), although surprising evidence from neighboring countries show the unlikely role of SSE in the spread of the disease (58). 

**4.5 Effective molecular screening of SARS-COV-2 and socioeconomic relations**

The *Coronaviridae* family is characterized by a positive-sense single-stranded RNA genome. Mouse hepatitis virus is a representative member of the family (41). Additionally, human hepatitis E virus also has a positive-sense single stranded RNA genome and shares a common evolution pathway with coronaviruses (40). Hepatitis-related incidents were described for SARS (59). The genetic recombination of these viruses within arbitrary intermediate hosts produced contagious strains that are extremely pathogenic to humans (40, 60). In this respect, the relation of SARS-COV genetic sequences isolated from human, civets, and bats permitted us to find the reason for such a dangerous epidemic, which affected people on a worldwide scale in 2003 (61). Moreover, the unpredictable epidemic of MERS-COV posed a serious risk to the health of communities worldwide. These underscored the necessity for further research of the virus epidemiology and pathophysiology to develop successful therapeutic and preventive medications against MERS-COV infection (62). While SARS-COV-2 is genetically and structurally connected with MERS-COV, it has its own exclusive structures which are responsible for its quick spread throughout the world (60). 

Specifically, variations in coronavirus pathogenicity within different species (63) make the understanding of SARS epidemics even more unclear through their capability to overcome the barrier for cross species transmission, which also alters their infectivity status (14, 64). As a result, boosting the pathogenic behavior of *coronavirus *strains, within species (65), and across species barriers (49), which is a reflection of their positive adaptation to rapid recombination events (49). The recent MERS epidemic revealed the tendency of the strain to genetically adapt and produce greater outbreaks (49) as occurred in SARS epidemic in 2003 (66). However, mainly for socioeconomic reasons, alarm signals were ignored until recently (67). A new phylogenetic analysis technique employed on clustered COVID-19 strains displayed a geographic variation preference in infectivity and pathogenesis (39). This is probably due to predominating strain’s tendency to cause an SSE as an outcome of a multi- factorial epidemic process presented in Figure 2 (23, 24). Marked SSEs for COVID-19 have already been fully characterized and warrant urgent investigation (23, 24). As presented in Tables 3 and 4, each way of transmission should be investigated. Heterogeneity of epidemic characteristics across nations (39) implies that in this way we may minimize coronavirus transmission. Therefore, salvation of national economic catastrophes will also be achieved in this way (66). Thus, the whole Biomedical Science machinery needs to perform targeted diagnosis of SSEs and share the obtained experience. Subsequently, central authorities will no longer need excessive non-specific contact measures, which will in turn normalize both societal and economic activities. 

**4.6 SSE-related large outbreaks and uncontrolled austerity**

On the other hand, improper understanding of how COVID-19 spreads resulted in societal imbalance due to arbitrary restriction of social and religious life including Holy Communion Cup. It has been consecutively demonstrated by expert research that the Holy Cup (Chalice) and the Holy Cloth are not sources or pathways, for potential spreading of infectious diseases including Human Immunodeficiency virus (HIV) (68), Hepatitis B virus (HBV) (69) as well as other communicable pathogens (70). Specifically, a review (69), considered other 129 relative studies. In this review, the possibilities that the shared communion cup can act as a vehicle for indirect transmission of human immunodeficiency virus, since it was detected in the saliva of infected individuals, was investigated. It was emphasized that although for bacterial contamination, the alcoholic content of the wine, the material that the cup is made of, or the practice of partially rotating the cup, cannot stop the occasional transmission of microbes, the microbial transmission was considerably reduced by the intervening use of a cloth to swab the lip of the cup between communicants. Notably, it was emphasized that transmission means not an obligatory inoculation or infection. Furthermore, it was also emphasized that out of the epidemiology of microbes transmitted via saliva, particularly for the transmission of the herpes viruses, the indirect transmission is rare, and indeed transmission is highly possible by other means than by the saliva. It was also emphasized that neither hepatitis B virus nor human immunodeficiency virus infection can be transmitted by saliva, rendering their indirect transmission also less likely by inorganic objects. Finally, the study concluded that no episode of disease transmission has ever been reported as a result of the shared communion cup use, and that there was not any scientific evidence that the communion cup practice should be abandoned due to the possible risk of spreading of any infection (71, 72). Likewise, Kingston et al. (68), by considering 44 relative papers, also concluded that there is no evidence that the Holy Communion Cup spreads infections. Moreover, more recent estimations also demonstrated that no infections have ever been observed as a result of religious rituals including Christian Common Communion chalice practice (70); whereas, data of previous studies implied that saliva could play a role in HBV transmission, are likely to be trivial (69). Similarly, recent evidence indicate that, although HBV DNA and HCV RNA can be discovered in the saliva of infected patients, they seem unlikely to transmit infection (72). It should be noted that, as in the case of coronavirus (73, 74), HBV also exists in many body fluids including saliva, nasopharyngeal fluid or tears by measures of qualitative and PCR methods (75). 

The detection of HBV DNA in saliva motivated our study group to investigate the potential viral transmission through the Holy Communion Cup. Two successive retrospective studies were conducted to investigate the role of Holy Communion as an independent risk factor of HBV dispersion. The first preliminary study included patients from our registry of those with chronic hepatitis B under entecavir (Jannis Kountouras-personal communication) treatment (76), and in the next step, the relative registry of another Department of the same Hospital was incorporated. Other parameters studied, the substantial independent categorical variable to evaluate our hypothesis was the patients’ occupation, thereby introducing two sub-groups; priests and non-priests. This classification was performed based on a standard active and perpetual exhibition (at least once weekly) of priests to many people’s saliva, as a part of the grounded process of the Holy Communion Cup. The control group comprised of the aggregate of Orthodox priests in Greece (10,338) and the rest general population (10,680,866) at that timeframe. Approval of the Institutional Ethics Committee was obtained and all predispositions of the Helsinki Declaration were fulfilled. The reservoir database did not include any personified information (name, ID number, etc.) and thus no informed consent was required. Pearson's chi-squared test with 1 degree of freedom was performed to evaluate whether there was a statistically significant difference between the frequencies of HBV infection in case and control groups and statistical significance was set at *p <0.05*.

The first single-centre registry included 71 patients and one (1.4%) of them was a priest. Chronic hepatitis B was significantly more frequent among non-priests compared to priests (x^2^ (1, N=71)=12.65, *p <0.05*). The extended sample (N=429) included the registry of another Department and an aggregate of four (0.93%) priests were diagnosed with chronic hepatitis B. Likewise, the chi-square test revealed that non-priest subjects were more likely to suffer from chronic hepatitis from HBV infection compared to priests (x^2^ (1, N=429) = 31, *p <0.001*). In conclusion, both of our analyses indicated a lower prevalence of HBV chronic hepatitis among priests when compared to other occupations. 

**Table 1:** Literature included from PubMed search for SSEs^*^ in relation to coronavirus outbreaks

**Literature included in review for SARS** ^^^ ** and MERS** ^!^ ** in relation to SSEs** ^*^			
Authors and Year		Type of article^**^	Study description
Chowell et al. (2015)(51)		Comparative research	Investigation of relation between SSEs for SARS and MERS transmission in nosocomial outbreaks
Al Tawfig et al. (2020) (38)		Commentary	Demonstration of a stochastic model of transmission of SARS virus
Shaw (2006) (55)		Perspective review	Implementing efficient intensive care practices to avoid hospital transmission
Chen et al. (2006) (96)		Original research Case control study	Investigation of SSE likelihood during hospital transmission
Sung et al. (2009) (97)		Original research Case control study	Investigation of SSEs occurring in hospital and prevention strategies
Li et al. (2004) (54)		Original research Case control study	Investigation of factors contributing to SSEs for prevention and control of disease
Riley (2003) (5)		Original research Cross sectional study	Analysis of SARS epidemiology in Hong Kong
Stein (2011) (98)		Perspective review	Analysis of SARS transmission leading to SSE
Gormely et al. (2017) (52)		Original research	Model for prevention of pathogen transmission via sanitary plumping systems
Lau (2004) (53)		Perspective review	Implementation of SSE containment with vaccination programs
**Literature included in review for COVID-19 in relation to SSEs**			
Cave (2020) (56)	Perspective review		Call for clear epidemiologic definition for SSEs
Xu (2020) (57)	Original research Retrospective cohort study		Analysis of SSEs during COVID-19 in China
Kwok (2020) (58)	Original research		Analysis of SSE influence in the nature of COVID-19 epidemic
Zhang (2020)(21)	Original research		Description of SSE importance in COVID-19 epidemic

^! ^Severe Acute Respiratory Syndrome; ^^ ^Middle East Respiratory Syndrome; ^&^Coronavirus Disease-19;^ *^Super Spread Events;^ **^When clearly indicated in article, the type of study is also mentioned.

**Table 2:** The search results of literature related to COVID-19& global impact due to SSEs^*^

**Search Item **	**Medline** **/PubMed**	Other electronic sources**	**Number of articles retrieved **	**Number of articles included **
SARS^!^, MERS^^^, and COVID-19 epidemic prevention	42	3589	139	17
SARS, MERS, and COVID-19 infectivity and pathogenicity	672	3812	145	18
Coronavirus SSE prevention	10	627	151	22
SSE, coronavirus crisis, and socio-economics	4	3181	89	11
Holy Cup religion and transmission of pathogens and SSEs	0	15	4	4
Coronavirus immunity and vaccination	73	20975	1175	9

^&^Coronavirus Disease-19; ^*^Super Spread Events; ** Science Direct, SciFinder, and Google Scholar; ^!^Severe Acute Respiratory Syndrome; ^^^Middle East Respiratory Syndrome

**Table 3:** Key clinical and laboratory screening functions to appropriately forecast, prevent, and confront SARS^!^ Coronavirus 2, and future coronavirus epidemic waves

**Specific Clinical and Laboratory Investigation**	**Validated techniques to be used**
**Forecasting of pre-symptomatic infection **	To estimate the probability of a major outbreak, use simulations of stochastic compartmental epidemic models. Use of diagnostic tests to detect asymptomatic susceptibility and pre-symptomatic infectivity
**Estimation of Super Spread Events of current and previous coronavirus epidemics **	Introduction of individual reproductive number. Integrated and computational analysis of the influence of individual variation by binomial distribution and use of branching process analysis of disease data**.**
**Genetic characterization of inpatient viral isolates to identify intermediate animal hosts facilitating the infection**	Next generation sequencing of samples and cultured viral isolates to obtain full sequence and phylogenetic analysis application.
**Environmental detection and continuous sewage monitoring**	RT-qPCR^#^ screening on sewage systems, vectorsو and potential air transmission. Autopsies and detection of serology conversion of potential vectors.
**Heptad repeat region screening for positive selection **	Computer simulation models to detect positive selection events e.g. *codeml branch-site *Test coupled with Bayes empirical Bayes procedure, and mixed effects model of evolution.
**Receptor recognition analysis of ACE-II** ^+^ ** to identify origin of cross-species and human to human transmissions coronaviruses**	Genetic sequencing and phylogenetic analysis of ACE-II to provide origin and efficiency of cross-species and human to human transmission and identification of intermediate hosts.

^!^Severe Acute Respiratory Syndrome; ^#^Reverse Transcriptase Quantitative Polymerase Chain reaction; ^+^Angiotensin-converting enzyme-II.

**Table 4:** Potential groups of coronavirus super spreaders within the human population^*^

**Population Group**	**Potential route of transmission**
Hepatitis B and C virus positive patients	Airborne
Pulmonary tuberculosis positive patients	Airborne
HIV^!^ positive patients	Airborne, urine & fecal-oral (98)
Patients receiving hemodialysis	Airborne (droplets by nebulizer) and fecal-oral
MRSA^#^* Staphylococcus aureus* acquisition	Constant Worn Glove Contact Transmission
Rhinovirus co-infections	Airborne
Gastrointestinal *(Salmonella enteritis)* co-infections	Fecal – oral
Frequent contact with wild animal reservoirs (including domestic animals) and birds^**^	Airborne and fecal – oral
Construction area workers	Air particles
Sewage system workers^***^	Fecal – oral

^*^In both community and hospital environments.

^**^Including slaughter houses, pet shops, animal and bird collectors and breeders, cow, and pig farmers.

^***^Including workers coming in contact with environment contamination.

^!^Human Immunodeficiency Virus, ^#^Methicillin Resistant *Staphylococcus aureus*.

**4.7 Coronavirus vaccination and relationship with SSEs**

Currently, vaccines for COVID-19 are in pre-clinical development, and no final clinical phase has been ended due the recent emergence of the disorder. Many global entities have stated their plans to produce a vaccine for COVID-19. According to the WHO, 41 candidate vaccines are being produced for COVID-19 as of March 13, 2020 (77). Importantly, for production of highly effective and safe COVID-19 vaccines, features such as the possibility of the induction of antigen-dependent enhancement (ADE) and additional severe opposing effects previously detected with SARS and MERS should be considered. ADE is a phenomenon that occurs when non-neutralizing antibodies against proteins of a virus increase, also increasing virus infectivity (78). In this regard, coronaviruses can escape the immunity provided by inactivated or recombinant protein vaccines via fast evolution (79). The problem with live attenuated vaccines is that the coronavirus can recover its virulence via serial passages in cell culture or *in vivo* (80). Moreover, vaccination in animals and humans could facilitate, rather than inhibit, the pathogenesis of the targeted viruses. This can be the consequence of an ADE phenomenon. This underlines a mechanism by which specific antibodies facilitate infection with the targeted virus, or cell-based augmentation, a process resulting in an allergic inflammatory response induced by immunopathology (81, 82).

**Many experimental SARS-CoV-1 vaccines have been formulated from whole inactivated viruses, due to their advantage of large-scale production, multiple epitope presentation and high conformation stability (** 83 **). One such vaccine uses viruses from AY71A217 strain of SARS-CoV-1, which are double inactivated using formalin and UV irradiation, the so-called double-inactivated virus (DIV) vaccine (** 84 **). Although DIV had initially been demonstrated to induce neutralizing antibodies and to protect against SARS-CoV-1 viral replication, both in tissue culture and in young mice, it soon became apparent that older mice suffered from vaccine-induced immune pathologies, including failure to contain viral replication, augmented clinical disease and associated symptoms, and increased inflammatory response and eosinophilic influx (** 84 **,** 85 **). In this respect, there is** **an overlap between the immunopathologic responses connected with coronavirus disease and vaccination, and the role of T helper (Th) 17 cells in immune augmentation and eosinophilic lung immunopathology; host Th17 polarized inflammatory reactions portray an important role in the pathophysiology of COVID-19 pneumonia and edema** **(**86**, **87**). Eosinophilic pathology, indicating increased pathogenesis and disease severity in the elderly, has been attributed to the nucleocapsid (N) protein, despite the incorporation of multiple SARS-CoV-1 antigens in the DIV (**82**, **84**). This is on grounds that the N protein is a strong modulator of innate immunity, also acting as an interferon antagonist, and therefore, it has the capability to induce inflammation with subsequent immune pathology in situations of heterologous viral challenge or in immune senescence, where patients fail to mount effective immune responses against the disease (**84**, **88**). The route of transmission is important to be established for SARS COV-2. As seen with other important infectious diseases of a) air borne transmission such as tuberculosis (**89**), b) orofecal transmission such as HEV (**90**) and c) blood transmission such as HBV and hepatitis D virus (**91**), even if efficient vaccination is established, understanding of SSEs is still important. Recent research data on the immune receptors used by coronaviruses, which reflect their ability to propagate in the human population, imply that complex immune reactions are responsible for a cell to cell transmission. In addition to ACE-II receptor, as is the case with SARS-COV, MERS-COV (**92**) and possibly for SARS-COV-2 (**92**, **93**), viruses use complex receptor recognition systems common to immunopathology damage mechanisms in coronavirus-infected individuals, which clearly define the clinical outcome (**94**). Therefore, application of vaccines that may interfere with antibody-mediated infection by coronaviruses (**95**) without true epidemiologic containment of coronaviruses, to restrict genetic adaptation events and inevitably producing an SSE, may be a miscellaneous attempt. However, synergy of SSE prevention measures with proper vaccination can provide a robust attempt for disease containment. **

## Limitations

This study aimed to perform a literature review. Although effort was made to decrease the risk of bias of results via double-blind screening of literature and employment of multiple electronic search engines, bias cannot be eliminated due to incomplete retrieval of identified research and biased estimations of included literature conclusions and methods used. Outcome of the study may also contain biased estimations originating from wrong interpretation of super spreading individuals in literature reviewed for SARS, MERS, and COVID-19 outbreaks. Although the importance of SSEs in COVID-19 was recognized by this study, more data from future accumulated epidemiology studies are needed to justify these findings. 

## Conclusions

Taken all together, management of SSEs is mandatory to yield efficient control over SARS-CoV-2. This is achievable through early diagnosis of pre/asymptomatic infected individuals within potential super spreading groups. Prevention of outbreaks is more essential, especially due to the lack of efficient vaccination and therapeutic protocols, which necessitates efficient monitoring, as SARS-COV-2 virus follows complex infectious patterns. The SARS-COV-2 epidemiological models that do not take SSEs into consideration seem to lead to confusing results with high uncertainty. SARS-CoV-2 causes prolonged “pandemics” through complex adaptation routes. Currently, in addition to the high technology utilized for diagnosis, clinical observation is indispensable to deeply comprehend SSEs and prohibit further outspread of COVID-19. Reference laboratories with efficient and accredited molecular and serological diagnosis must be inter-linked between countries. All these parameters could contribute to avoiding a second blind unjustified response that characterized the first COVID-19 pandemic spread. Understanding the epidemiology of COVID-19 through SSEs could be preventive for future epidemics. A systematic meta-analysis research methodology, when COVID-19 epidemiology data accumulate further, would be advisable to confirm the conclusions of this study.

## Ethics approval and consent to participate

This study did not involve the participation of any humans or animals as it was based only on literature research. 

## Consent for publication

All authors agree to publish this manuscript. 

## Availability of data and materials

All data used for this manuscript are available upon request 

## Competing interests

All authors declare that they have no competing interests. 

## Funding

No funding or grant was received for this study. 

## Abbreviations

SARS: Severe acute respiratory syndrome.

SARS-COV-2: Severe acute respiratory syndrome coronavirus -2.

SSEs: Super spread events.

COVID-19: Coronavirus disease 2019.

SADS: Swine acute diarrhea syndrome.

R_o_: Basic reproduction number.

IQR: Interquartile range.

MERS-COV: Middle East Respiratory Syndrome coronavirus.

HIV: Human Immunodeficiency virus

HBV: (Human) Hepatitis B virus

HCV: (Human) Hepatitis C virus

ADE: Antigen dependent enhancement

DIV: Double Inactivated virus

Th: T helper (cell)

RT-qPCR: Reverse transcriptase quantitative polymerase chain reaction

ACE-II: Angiotensin converting enzyme II

MRSA: Methicillin resistant *Staphylococcus aureus*
